# Pathogenic germline variants in *BRCA1*/*2* are associated with a decreased number of hypomethylated rDNA copies

**DOI:** 10.1186/s13058-026-02387-5

**Published:** 2026-09-25

**Authors:** Sarah Kießling, Jana Durackova, Alina Michler, Alva B. C. Geisen, Natalia Santana Acevedo, Thomas Haaf

**Affiliations:** 1 Institute of Human Genetics, Julius Maximilians University, 97074 Würzburg, Germany; 2 Institute of Clinical Genetics and Genomic Medicine, University Clinics, 97080 Würzburg, Germany

**Keywords:** Absolute rDNA copy number, Hypomethylated rDNA copy number, *BRCA1*/*2* pathogenic variant, Deep bisulfite sequencing, Droplet digital PCR, Familial breast and ovarian cancer, rDNA methylation

## Abstract

**Background:**

Little is known about the phenotypic impact of the enormous rDNA copy number (CN) variation among individuals. The rDNA CN appears to be stable across different tissues of the same individual, whereas many tumors exhibit consistent rDNA CN changes, compared to healthy tissue. Herein, we have analyzed rDNA CN in blood of healthy carriers of *BRCA1*/*2* pathogenic variants (PV), who have a high risk of developing breast and ovarian cancer.

**Methods:**

Using a combination of digital droplet PCR and deep bisulfite sequencing we have determined the absolute rDNA CN and the number of copies with a hypomethylated (≤ 10%) promoter region in healthy *BRCA1* (*N* = 90) and *BRCA2* (*N* = 71) PV carriers as well as in PV-negative controls (*N* = 146).

**Results:**

Absolute CN was comparable between groups. However, there was a significant and an almost significant difference, respectively, in hypomethylated rDNA CN between females carrying a *BRCA1* (*P* = 0.023) or *BRCA2* PV (*P* = 0.085), compared to controls. The effect appeared to be restricted primarily to younger women. PV carriers younger than 45 years (69 *BRCA1*, 47 *BRCA2*) exhibited approximately 10 hypomethylated copies less (*P* = 0.015 for *BRCA1* and 0.150 for *BRCA2*) than age-matched controls (*N* = 98).

**Conclusions:**

Our results link germline *BRCA1*/*2* PV to rDNA epigenetic regulation. We propose that the decreased number of hypomethylated, presumably active CN may contribute to cancer susceptibility in *BRCA1*/*2* PV carriers, preceding tumor development.

**Supplementary Information:**

The online version contains supplementary material available at https://doi.org/10.1186/s13058-026-02387-5.

## Background

Ribosomes are composed of ribosomal RNAs and proteins; they are essential for protein translation, and consequently for all cellular processes. The 45 S rDNA transcription units (TU) which encode the 18 S, 5.8 S and 28 S rRNAs are tandemly arrayed on the acrocentric short arms [1]. The absolute number of rDNA TU in the diploid human genome can vary from < 200 to > 1,000 among normal individuals [2–5].

In our previous studies we used droplet digital PCR (ddPCR) and deep bisulfite sequencing (DBS) to determine absolute rDNA copy number (CN) as well as the number of presumably active copies with a hypomethylated (≤ 10%) promoter region. With some notable exceptions absolute and even more hypomethylated rDNA CN were comparable in different tissues of the same individual [6]. In blood DNA samples and by extrapolation in other somatic tissues, the number of hypomethylated copies decreased, whereas the number of methylated (> 10%) copies increased with age [7]. We propose that hypomethylated rDNA copies are presumably active. The decreasing hypomethylated rDNA CN and activity with age may interfere with ribosome production and protein synthesis.

Active rDNA CN affects ribosome biogenesis and consequently cell metabolism, growth, and proliferation during development, aging and disease [8–10]. Because repetitive and CpG-rich regions are difficult to analyze by current genomic sequencing technologies, surprisingly little is known about the biological consequences of the enormous interindividual variation of rDNA CN. Accumulating evidence suggests a role for rDNA CN as a modulating factor in multifactorial traits [11], including male infertility [12], schizophrenia [13], body mass index [14], blood cell composition and kidney function [5]. Recently, it has been shown that in newborns absolute rDNA CN is associated with methylation changes of individual CpGs throughout the genome, which may influence cellular functions and disease trajectories later in life [15]. In addition to CN, germline sequence variation of rDNA has been associated with altered rRNA structure and multiple human complex phenotypes, in particular body size traits [16].

Compared to healthy tissue, many cancers display consistent rDNA methylation changes [8]. rDNA instability resulting in gains or losses of rDNA CN in tumor compared to healthy tissue is thought to occur at disease onset and progression [17]. In this study, we investigated rDNA CN variation in healthy individuals with a genetic tumor predisposition syndrome. Approximately 5–10% of all breast cancers are hereditary, most commonly caused by germline pathogenic variants (PV) in the tumor suppressor genes *BRCA1* and *BRCA2*, which play a crucial role in DNA double-strand break repair [18]. Heterozygous females with a germline PV have a life-long risk of 50–70% for developing breast cancer and of 10–50% for developing ovarian cancer (https://www.cdc.gov/breast-ovarian-cancer-hereditary/causes/index.html). Here we compared absolute and hypomethylated rDNA CN in healthy *BRCA1*/*2* PV carriers and PV-negative controls.

## Methods

### Study samples

The study on anonymized diagnostic samples was approved by the Ethics Committee at the Medical Faculty of Würzburg University (nos 149/20_z-sc and 20220718 01). The *BRCA1*/*2* cohorts consisted of 90 unrelated healthy women carrying a PV in *BRCA1* and 71 females with a PV in *BRCA2* (Supplementary Table 1). Variants were classified according to the criteria of the American College of Medical Genetics and Genomics and the Association for Molecular Pathology (ACMG/AMP) [19] (ClinGen Internal VCEP ACMG/AMP specifications for *BRCA1* and *BRCA2*, Version 1.2.0). With exception of two likely pathogenic *BRCA1* variants (in samples 50 and 229), all variants were classified as pathogenic (IARC class 5). The control group consisted of 146 age-matched women without PV in predictive genetic testing. The age ranged from 18.2 to 78.5 years.

To study possible age effects on rDNA CN, we formed subgroups of 214 (69 *BRCA1*, 47 *BRCA2*, and 98 controls) younger (< 45 years of age) and 93 (21 *BRCA1*, 24 *BRCA2*, and 48 controls) older (> 45 years) women. The 45-year threshold was more or less arbitrarily defined to ensure sufficient sample size in both groups. Our cohort of healthy *BRCA1*/*2* PV carriers mainly consisted of younger women, who opted for predictive genetic testing, whereas overall most breast cancers manifest after the age of 45 years.

DNA was prepared from EDTA blood samples by the classical salting out method. The DNA concentration and purity were determined by the Qubit dsDNA BR Assay system kit (Invitrogen, Karlsruhe, Germany) and the NanoDrop 2000c spectrophotometer (ThermoScientific, Waltham, MA, USA). Following ddPCR, all DNA samples underwent bisulfite conversion using the EpiTect Fast 96 Bisulfite kit (Qiagen, Hilden, Germany).

### Droplet digital PCR 

ddPCR is a quantitative PCR method that can determine the number of a given DNA molecule, i.e. rRNA genes in a DNA sample without using a standard curve [20]. ddPCR primers (Supplementary Table 2) for 28 S rDNA and the human TATA-box binding protein (*TBP*) gene (internal reference) were adopted from the literature [21]. ddPCR for absolute rDNA counting was performed according to our previously published protocol [7, 22].

### Deep bisulfite sequencing (DBS)

DBS primers (Supplementary Table 3) were designed for the human upstream control element (UCE) and core promoter (CP) region containing 25 contiguous CpGs and an A/G variant (GRCh38; chr21:8.205.892) [23]. Overall > 95% of reads represented the major G allele. To avoid an effect of genetic variation on our methylation results, only reads representing the major variant were used for downstream analyses.

rDNA library preparation was performed exactly as described in our previous studies [7, 22]. Following Illumina’s (San Diego, California, USA) recommendations for sequencing low diversity libraries with the NextSeq 2000, the rDNA library was spiked with 20% PhiX. Sequencing with the Reagent Kit P1 (300 cycles) (Illumina) yielded 150 bp paired-end reads, which were processed by the Illumina BCL Convert software version 4.2.7. Only reads with an overall bisulfite conversion rate > 95% were considered for downstream processing of FASTQ files using Amplikyzer2 software [24].

### Statistical analysis

Both descriptive and inferential statistical analyses were conducted using IBM SPSS version 29. The number of presumably active rDNA copies with ≤ 10% promoter methylation was calculated by multiplying the absolute CN (from ddPCR) with the percentage of reads (from DBS) with 0–10% methylation in the UCE/CP region. The Kolmogorov-Smirnov and Shapiro-Wilk tests were used to determine data distribution. Group comparisons, based on data distribution, were conducted using the non-parametric Mann-Whitney U test. Spearman’s correlations were used to determine the relationship between two continuous variables, such as hypomethylated CN and age. *P* values < 0.05 were considered statistically significant.

## Results

### Absolute rDNA CN in BRCA1/2 mutation carriers and controls

**Fig. 1:**
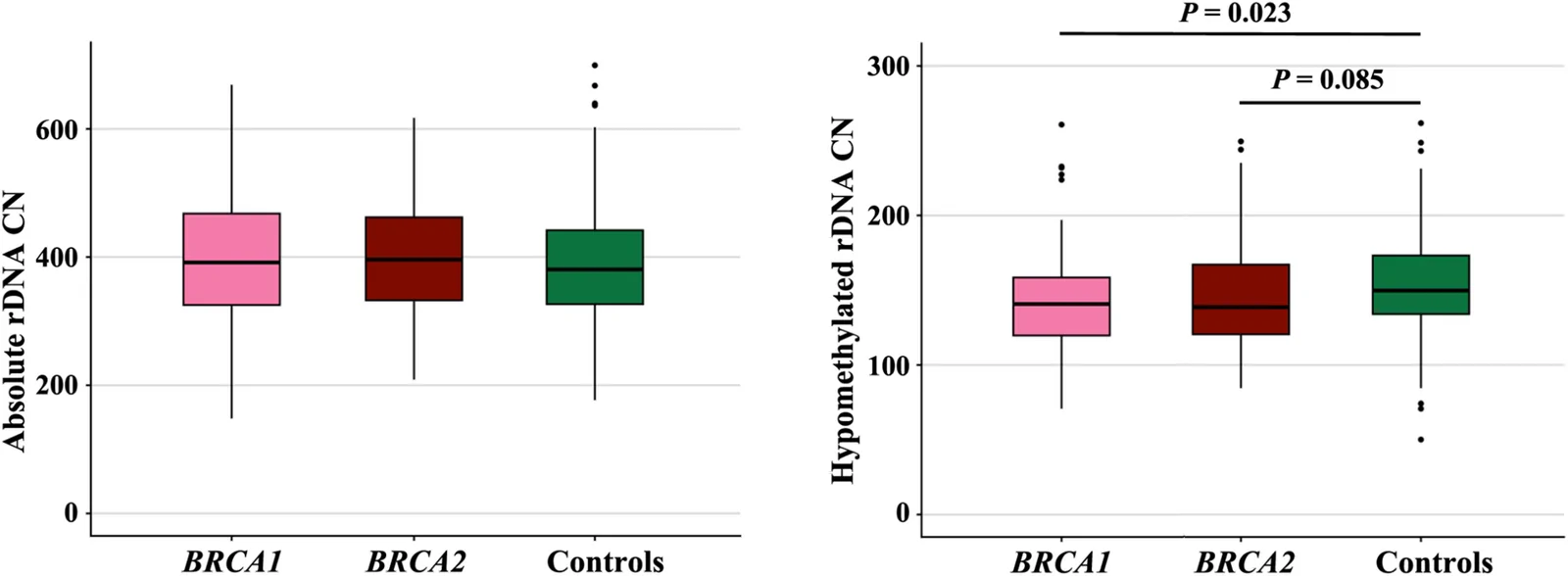
Comparison of absolute (left diagram) and hypomethylated rDNA CN (right) between *BRCA1* (*N* = 90) PV, *BRCA2* (*N* = 71) PV carriers and PV-negative controls (*N* = 146). The median is presented by a horizontal line. The bottom of the box indicates the 25th and the top the 75th percentile. Outliers are indicated by black dots. There are no significant between-group difference in absolute CN. In contrast, hypomethylated CN is significantly lower (Mann-Whitney U test *P* = 0.023) in *BRCA1* (142 ± 35) and almost significantly lower (*P* = 0.085) in *BRCA2* (145 ± 36) PV carriers, compared to controls (152 ± 37). There is no difference in hypomethylated CN between *BRCA1* and *BRCA2*

Using ddPCR, we determined absolute rDNA CN in 90 *BRCA1* PV carriers, 71 *BRCA2* PV carriers, and 146 PV-negative controls. All measurements were performed in duplicates. For all samples, there was a strong correlation (Spearman’s ρ = 0.96; *P* < 0.001) between the first and the second replicate (Supplementary Fig. 1A). The absolute rDNA CN was 397 ± 104 (median 392; range 148–669) in *BRCA1* PV carriers, 400 ± 99 (396; 209–617) in *BRCA2* PV carriers, and 393 ± 96 (380; 177–699) in controls. There was no significant difference between groups (Mann-Whitney U test *P* = 0.56 for *BRCA1* vs. controls; *P* = 0.43 for *BRCA2* vs. controls; and *P* = 0.78 for *BRCA1* vs. *BRCA2*) (Fig. 1, left diagram).

### Hypomethylated rDNA CN in BRCA1/2 PV carriers and controls

Using a combination of ddPCR and DBS, we determined the number of copies with a hypomethylated promoter region. Again, there was a strong correlation (ρ = 0.84; *P* < 0.001) between technical replicates, including ddPCR and DBS (Supplementary Fig. 1B). The hypomethylated rDNA CN was 142 ± 35 (median 141, range 71–261) in *BRCA1* PV carriers, 145 ± 36 (139, 85–250) in *BRCA2* PV carriers, and 152 ± 37 (150, 50–262) in controls. There was a significant difference between *BRCA1* and controls (Mann-Whitney U test *P* = 0.023) and an almost significant difference between *BRCA2* and controls (*P* = 0.085), but not between *BRCA1* and *BRCA2* (*P* = 0.81) (Fig. 1, right diagram).

**Fig. 2:**
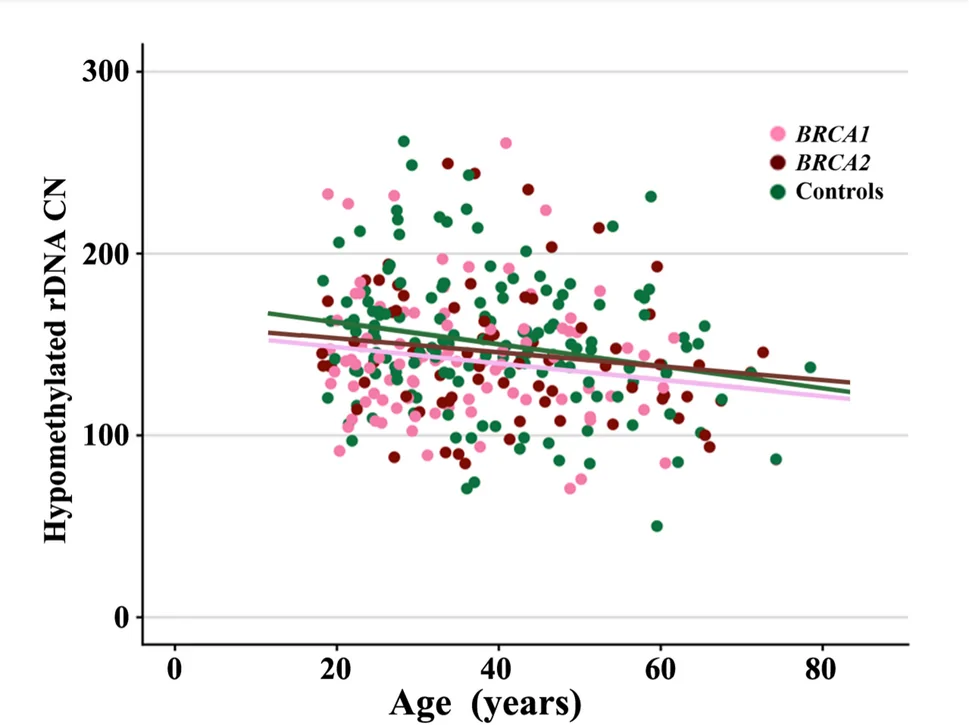
Relationship between hypomethylated rDNA CN and age. Pink dots represent 90 *BRCA1* PV carriers, brown dots 71 *BRCA2* PV carriers, and green dots 146 age-matched controls. In controls hypomethylated CN significantly decreased with age (Spearman’ s ρ = − 0.21, *P* = 0.013), whereas *BRCA1*/*2* PV carriers did not show a significant correlation between hypomethylated CN and age (ρ = − 0.09, *P* = 0.40 for *BRCA1* and ρ = − 0.17, *P* = 0.15 for *BRCA2*)

**Fig. 3:**
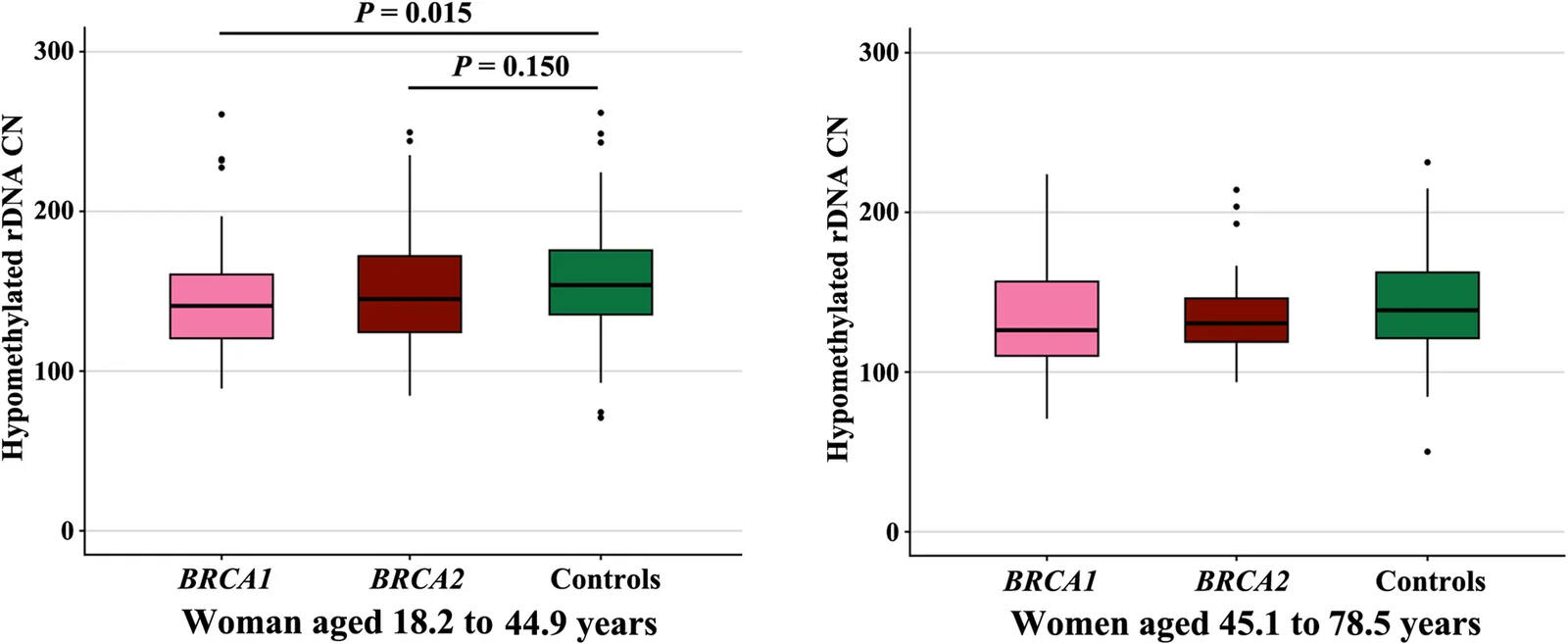
Comparison of hypomethylated rDNA CN in *BRCA1* PV carriers, *BRCA2* PV carriers, and controls. The median is presented by a horizontal line. The bottom of the box indicates the 25th and the top the 75th percentile. Outliers are indicated by black dots. The box plots in the left diagram represent 214 women younger than 45 years (69 *BRCA1*, 47 *BRCA2*, and 98 controls). There is a significant (*P* = 0.015) difference between *BRCA1* PV carriers (145 ± 34) and controls and borderline significant difference (*P* = 0.150) between *BRCA2* PV carriers (148 ± 38) and controls (157 ± 37). The right diagram represents 93 women older than 45 years (21 *BRCA1*, 24 *BRCA2*, and 48 controls). There are no significant between-group differences

Consistent with earlier studies [7], the hypomethylated CN in controls was significantly decreasing with age (Spearman’ s ρ = − 0.21, *P* = 0.013). Surprisingly, this age effect was not seen in *BRCA1* PV (ρ = − 0.09, *P* = 0.40) and *BRCA2* PV (ρ = − 0.17, *P* = 0.15) carriers (Fig. 2). When comparing the linear regression models for *BRCA1*, *BRCA2* and controls, the reduced hypomethylated CN in *BRCA1*/*2* PV carriers was most noticeable in younger women. Therefore, we analyzed hypomethylated CN in women younger (*N* = 214) and older than 45 years (*N* = 93) separately. In the younger women (Fig. 3, left diagram), there was a significant (*P* = 0.015) difference in hypomethylated CN between *BRCA1* PV carriers (145 ± 34, median 141, range 89–261) and controls (157 ± 37, 154, 71–262) and a borderline significant difference (*P* = 0.150) between *BRCA2* PV carriers (148 ± 38, 145, 84–250) and controls. There was no difference (*P* = 0.59) between *BRCA1* and *BRCA2*. In women older than 45 years (Fig. 3, right diagram), there were no significant differences (*P* = 0.29, 0.38 and 0.90, respectively) between *BRCA1* (131 ± 38, 126, 71–224), *BRCA2* (137 ± 31, 131, 94–214), and controls (141 ± 35, 139, 50–231).

## Discussion

### rDNA methylation and transcription

Previously, we have shown that both in somatic tissue [7] and germ cells [25] the rDNA CN with a hypomethylated (≤ 10%) promoter decreased and the CN with a methylated (10–100%) promoter increased with age, consistent with an age-related loss of hypomethylated rDNA copies. Genes with a hypomethylated promoter region are generally thought to be active [26, 27]. The higher an individual’s absolute CN, the higher the number of methylated copies. One plausible explanation is that in individuals with high absolute CN the excess copies are inactivated by promoter methylation. Thus, promoter methylation compensates at least to some extent for high absolute CN, ensuring comparable numbers of hypomethylated, presumably active CN in individuals with low (< 200) and high (> 600) absolute CN, respectively [6, 7].

A recent study using immunofluorescence in situ hybridization showed that the activity of rDNA arrays consistently varied between the different acrocentric chromosomes of an individual. Active arrays had low promoter methylation and were positive for proteins involved in rDNA transcription and ribosome biogenesis. Inactive arrays displayed high promoter methylation and lacked UBF and TREACLE proteins. Importantly, silent arrays could be reactivated by demethylation [28]. Although we do not really know which methylated CpG density in the promoter region is required for epigenetic rDNA silencing, it is plausible to assume that the number of copies with a hypomethylated (≤ 10%) promoter may be a good surrogate marker for rDNA transcriptional activity.

### Haploinsufficiency-induced effects of BRCA1/2

*BRCA1* and *BRCA2* are tumor suppressor genes which are essential for maintaining genome integrity in all cells of the body [18]. However, beyond the classical two-hit hypothesis, *BRCA1* haploinsufficiency in epithelial cells leads to CN alterations and senescence, even in the presence of an intact second allele [29, 30]. It has been proposed that cells, in particular epithelial cells with a heterozygous PV already exhibit an increased susceptibility to acquire additional genomic alterations [31]. Our results demonstrate comparable absolute rDNA CN in blood cells of *BRCA1*/*2* PV carriers and controls. This is consistent with the view that genomic instability of rDNA, which is a hallmark of metastatic breast cancer [17], does not precede tumor development, but occurs during tumor progression. In contrast, our study demonstrates a minor but significantly lower hypomethylated rDNA CN in healthy *BRCA1*/*2* PV carriers than in controls, linking *BRCA1*/*2* germline PV to rDNA epigenetic regulation. These epigenetic changes which are likely to affect rRNA expression may already occur early in life in heterozygous cells of healthy *BRCA1*/*2* PV carriers.

Previously we have shown that hypomethylated, presumably active CN remains stable from birth to sexual maturity and then is decreasing with age [22]. This supports the hypothesis that aging begins after reaching the reproductive phase [32]. An epigenetic clock based on increasing rDNA methylation with age appears to be conserved in different mammalian species and tissues [33, 34]. *BRCA1*/*2* PV carriers are endowed with a reduced number of hypomethylated rDNA copies at young age (18–45 years), whereas beyond the age of 45 years this difference between PV carriers and controls is fading out. It is tempting to speculate that at the level of epigenetic rDNA regulation, *BRCA1/2* PV carriers exhibit premature aging.

A low hypomethylated, presumably active rDNA CN at younger age may contribute to the increased risk for breast and several other cancers in healthy *BRCA1*/*2* PV carriers. Another line of evidence comes from Robertsonian translocation carriers, the most common (1 in 800) chromosome rearrangement in healthy individuals. The fusion of two acrocentric chromosomes involves the loss of two (of a maximum of 10) rDNA arrays on the acrocentric short arms. The reduced rDNA CN in Robertsonian translocation carriers is associated with a significantly increased cancer risk, including childhood leukemia, non-Hodgkin lymphoma and breast cancer [35, 36]. This supports the view that a low rDNA CN is associated with an increased cancer risk, also in the absence of a predisposing *BRCA1*/*2* mutation.

### Limitations

The activity of rRNA genes is epigenetically regulated by DNA methylation [26, 28] and the promoter methylation level is crucial for switching a gene from active to inactive [27]. However, in addition to rDNA methylation, modifications of RNA polymerase I factors, chromatin alterations, and lncRNAs may also play a role in the regulation of rDNA expression [37, 38]. Since we do not have RNA samples or cell lines of our study subjects we cannot study the functional consequences, i.e. whether the hypomethylated copies are transcribed. Moreover, it is difficult to prove by functional experiments, which methylated CpG density is required for epigenetic silencing and there may not be a sharp threshold anyways. So far [6, 7] we have only circumstantial evidence for transcriptional activity of hypomethylated rDNA copies.

It is important to mention that on average *BRCA1*/*2* PV carriers had 10 hypomethylated copies less than controls, but CN was still within the normal range of CN variation. Consistent with our previous analyses [6], at least 50 hypomethylated, presumably active rDNA copies may be sufficient for normal cell function and development. Overall, the effect size of reduced hypomethylated rDNA CN was small and appeared to be restricted primarily to younger women. rDNA methylation and activity may be only one of multiple factors contributing to an increased cancer risk in PV carriers. Phenotypic manifestation of breast cancer may occur when the number of epigenetic and other factors exceeds a critical threshold. Apart from genetic variants and age, both breast cancer risk (https://www.cdc.gov/breast-cancer/risk-factors/index.html) and DNA methylation are influenced by numerous factors, including physical activity [39], diet [40], body mass index [41], hormones [42], alcohol drinking [43], smoking [44], and other environmental exposures [45]. Due to our study design with anonymized DNA samples, none of these possible confounding factors could be taken into account.

At the level of the individual, rDNA CN cannot be associated with specific functional consequences or be used as a predictive marker for cancer risk. Consistent with a multifactorial disease model, the effects of reduced hypomethylated rDNA CN and by extrapolation activity can only be seen at the population level. Multicenter studies including confounding factors on a larger number of individuals are needed to replicate the association between *BRCA1*/*2* haploinsufficiency and a low number of hypomethylated, presumably active rDNA copies. Because haploinsufficiency-induced effects may be at least to some extent cell type-specific [29, 30], rDNA CN variation and activity should also be analyzed in breast epithelial cells.

One special feature of our study is that we have analyzed healthy *BRCA1*/*2* PV carriers who have not (yet) developed cancer. Hypomethylated, presumable active rDNA CN was modestly reduced in *BRCA1*/*2* PV carriers. The epigenetic chances observed are associated with and may contribute to cancer susceptibility. In patients who have already developed cancer dramatic methylation [8] and rDNA CN changes [17] are caused during malignant transformation. Moreover, cancer treatment (radio- and chemotherapy) does not only affect tumor cells but also healthy tissues, including blood. This would render the detection of subtle epigenetic signatures associated with a cancer susceptibility syndrome(s) difficult or even impossible.

## Supplementary Information

Below is the link to the electronic supplementary material.

Supplementary Material 1

## Data Availability

All data are contained in the manuscript and its supplements.
