# Supplementary material for: Pathogenic germline variants in BRCA1/2 are associated with a decreased number of hypomethylated rDNA copies
Source: Breast Cancer Res. 2026 Sep 25;28:161. doi: 10.1186/s13058-026-02387-5 (PMC13615621; doi:10.1186/s13058-026-02387-5)
Supplement: Supplementary file 1 [file 13058_2026_2387_MOESM1_ESM.pdf]

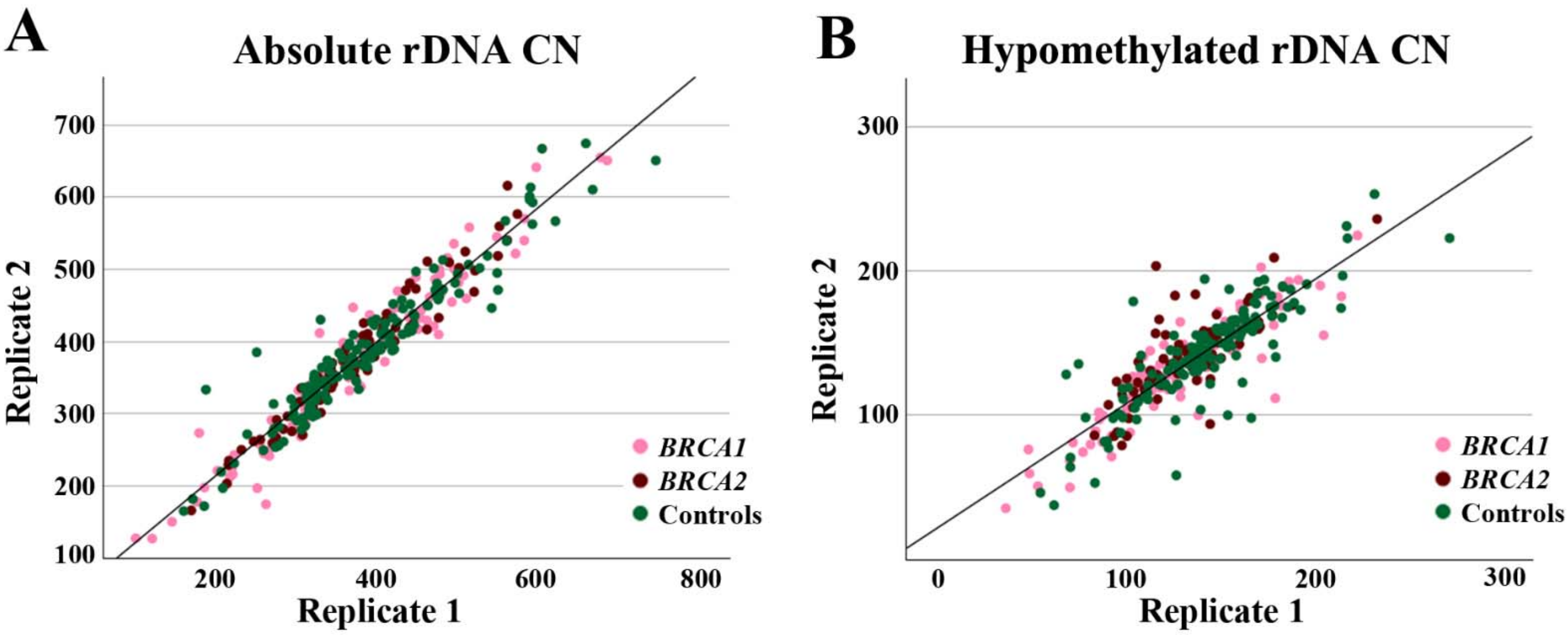

Supplementary Fig. 1 Duplicate measurements of (A) absolute CN (Spearman's ρ = 0.96; $P < 0.001$) and (B) hypomethylated CN (ρ = 0.84; $P < 0.001$) show a strong correlation between technical replicates. Pink dots represent *BRCA1* PV carriers, brown dots *BRCA2* PV carriers, and green dots age-matched controls.

Supplementary Table 1

Study samples: clinical and experimental parameters

Sample ID	Absolute CN	Active CN	IARC class	Pathogenic variant
BRCA1				
4	451.2	140.7	5	BRCA1: c.5333-1G>C
7	486.3	152.8	5	BRCA1: c.5266dup p.(Gln1756Profs*74)
9	437.1	181.5	5	BRCA1: c.5144G>A p.(Ser1715Asn)
10	397.9	130.5	5	BRCA1: c.2411_2412del p.(Gln804Leufs*5)
11	414.3	112.1	5	BRCA1: c.3193dup p.(Asp1065Glyfs*2)
12	346.7	177.7	5	BRCA1: c.5177_5180del p.(Arg1726Lysfs*3)
14	280.4	125.9	5	BRCA1: c.3481_3491del p.(Glu1161Phefs*3)
15	337.8	136.1	5	BRCA1: Ex 8 del
17	666.8	115.6	5	BRCA1: c.3481_3491del p.(Glu1161Phefs*3)
18	427.3	112.9	5	BRCA1: c.4689C>G p.(Tyr1563*)
19	436.2	121.6	5	BRCA1: c.3403C>T p.(Gln1135*)
20	547.4	140.8	5	BRCA1: c.3700_3704del p.(Val1234Glnfs*8)
21	371.0	164.5	5	BRCA1: c.4035del p.(Glu1346Lysfs*20)
23	392.3	142.9	5	BRCA1: c.310del p.(Ser104Alafs*15)
24	413.3	75.9	5	BRCA1: c.3578dup p.(Thr1194Hisfs*25)
25	349.2	143.4	5	BRCA1: c.3756_3759del p.(Ser1253Argfs*10)
26	254.3	156.7	5	BRCA1: c.5266dup p.(Gln1756Profs*74)
27	233.0	126.2	5	BRCA1: c.4986+3G>C
28	492.0	115.0	5	BRCA1: c.4349del p.(Ser1450*)
29	263.3	144.0	5	BRCA1: c.2411_2412del p.(Gln804Leufs*5)
30	378.8	167.8	5	BRCA1: c.5266dup p.(Gln1756Profs*74)
31	373.6	167.1	5	BRCA1: c.5351del p.(Val1784Aspfs*9)
32	620.1	70.8	5	BRCA1: c.329dup p.(Glu111Glyfs*3)
33	363.6	260.7	5	BRCA1: Ex 8 del
34	401.7	106.9	5	BRCA1: c.2411_2412del p.(Gln804Leufs*5)
35	324.5	157.2	5	BRCA1: c.4675+1G>A
36	536.9	114.1	5	BRCA1: c.5194-12G>A
37	273.7	141.2	5	BRCA1: c.3756_3759del p.(Ser1253Argfs*10)
38	446.5	170.8	5	BRCA1: c.3485del p.(Asp1162Valfs*48)
39	474.3	197.0	5	BRCA1: c.68_69del p.(Glu23Valfs*17)
40	317.3	127.0	5	BRCA1: c.5503C>T p.(Arg1835*)
41	364.9	93.7	5	BRCA1: c.4675+1G>A
42	308.7	123.3	5	BRCA1: c.3700_3704del p.(Val1234Glnfs*8)
50	328.6	148.0	4	BRCA1: c.5288G>A p.(Gly1763Glu)
224	287.3	84.8	5	BRCA1: c.4675+1G>A
225	472.6	153.6	5	BRCA1: c.4986+6T>G
226	668.9	179.4	5	BRCA1: Ex 21-24 del
227	366.9	139.1	5	BRCA1: c.3661G>T p.(Glu1221*)
228	479.7	135.0	5	BRCA1: c.5291T>C p.(Leu1764Pro)
229	256.8	153.3	4	BRCA1: c.5213_5215del p.(Gly1738del)

230	311.3	86.6	5	BRCA1: c.5266dup p.(Gln1756Profs*74)
231	485.8	120.5	5	BRCA1: c.2683_2686del p.(Gln895Valfs*104)
233	365.8	126.2	5	BRCA1: c.2410C>T p.(Gln804*)
235	460.7	150.3	5	BRCA1: c.66dup p.(Glu23Argfs*18)
237	302.9	148.9	5	BRCA1: Ex 22 del
238	339.7	119.7	5	BRCA1: c.2477_2478del p.(Thr826Argfs*4)
239	327.5	146.3	5	BRCA1: c.181T>G p.(Cys61Gly)
240	489.0	160.5	5	BRCA1: c.5291T>C p.(Leu1764Pro)
242	370.6	163.3	5	BRCA1: c.3481_3491del p.(Glu1161Phefs*3)
243	385.5	184.2	5	BRCA1: c.5266dup p.(Gln1756Profs*74)
245	443.6	118.4	5	BRCA1: c.181T>G p.(Cys61Gly)
246	391.8	139.1	5	BRCA1: c.181T>G p.(Cys61Gly)
247	404.5	141.7	5	BRCA1: c.5503C>T p.(Arg1835*)
248	434.7	231.8	5	BRCA1: c.4936del p.(Val1646Serfs*12)
249	498.5	191.8	5	BRCA1: c.2411_2412del p.(Gln804Leufs*5)
250	463.8	150.9	5	BRCA1: Ex 17 del
251	389.0	89.1	5	BRCA1: c.5406+5G>C
252	515.9	167.4	5	BRCA1: c.5503C>T p.(Arg1835*)
253	298.6	147.4	5	BRCA1: c.4741G>T p.(Glu1581*)
310	322.1	232.7	5	BRCA1: c.3296del p.(Pro1099Leufs*10)
311	221.5	128.5	5	BRCA1: c.4675+1G>A
312	192.0	91.4	5	BRCA1: c.5266dup p.(Gln1756Profs*74)
313	446.0	108.9	5	BRCA1: c.5266dup p.(Gln1756Profs*74)
314	211.8	139.0	5	BRCA1: c.4183C>T p.(Gln1395*)
315	469.3	178.1	5	BRCA1: c.5266dup p.(Gln1756Profs*74)
316	271.1	137.1	5	BRCA1: c.2411_2412del p.(Gln804Leufs*5)
320	449.2	107.9	5	BRCA1: c.3700_3704del p.(Val1234Glnfs*8)
321	266.3	142.7	5	BRCA1: c.5266dup p.(Gln1756Profs*74)
322	391.3	119.4	5	BRCA1: Ex1-7 del
323	148.2	102.4	5	BRCA1: c.5291T>C p.(Leu1764Pro)
324	561.9	110.5	5	BRCA1: c.181T>G p.(Cys61Gly)
325	318.5	130.0	5	BRCA1: c.1687C>T p.(Gln563*)
326	411.5	146.2	5	BRCA1: c.952_1015del p.(His318Argfs*2)
327	443.6	140.6	5	BRCA1: c.4986+6T>G
328	577.1	192.7	5	BRCA1: c.4689C>G p.(Tyr1563*)
329	256.5	158.4	5	BRCA1: c.53T>C p.(Met18Thr)
330	395.4	158.6	5	BRCA1: c.1127del p.(Asn376Ilefs*18)
331	547.4	223.8	5	BRCA1: c.1687C>T p.(Gln563*)
332	368.3	142.9	5	BRCA1: c.3481_3491del p.(Glu1161Phefs*3)
333	443.6	119.8	5	BRCA1: c.2338C>T p.(Gln780*)
334	328.4	158.9	5	BRCA1: c.5266dup p.(Gln1756Profs*74)
335	371.5	108.5	5	BRCA1: c.3485del p.(Asp1162Valfs*48)
336	375.5	110.2	5	BRCA1: c.5096G>A p.(Arg1699Gln)
345	469.2	140.8	5	BRCA1: c.2477_2478del p.(Thr826Argfs*4)
346	550.1	227.4	5	BRCA1: c.2410C>T p.(Gln804*)
347	485.7	104.5	5	BRCA1: c.4689C>G p.(Tyr1563*)
348	244.6	178.0	5	BRCA1: c.4183C>T p.(Gln1395*)

352	537.4	129.2	5	BRCA1: c.3661G>T p.(Glu1221*)
353	499.4	119.9	5	BRCA1: c.798_799del p.(Ser267Lysfs*19)
BRCA2				
52	443.7	170.2	5	BRCA2: c.5146_5149del p.(Tyr1716Lysfs*8)
53	461.2	118.0	5	BRCA2: c.7976G>A p.(Arg2659Lys)
54	415.3	235.2	5	BRCA2: c.1813dup p.(Ile605Asnfs*11)
55	293.1	194.1	5	BRCA2: c.9257-1G>C
56	325.8	120.1	5	BRCA2: c.6591_6592del p.(Glu2198Asnfs*4)
57	285.3	182.6	5	BRCA2: c.1405_1406del p.(Asp469*)
58	265.0	166.6	5	BRCA2: c.7758G>A p.(Trp2586*)
59	362.0	173.8	5	BRCA2: c.7316del p.(Gly2439Aspfs*30)
60	271.6	145.2	5	BRCA2: c.7976G>A p.(Arg2659Lys)
61	551.5	140.4	5	BRCA2: c.6514_6515del p.(Ser2172Thrfs*3)
62	404.8	138.2	5	BRCA2: c.6591_6592del p.(Glu2198Asnfs*4)
63	289.6	130.9	5	BRCA2: c.6531dup p.(His2178Serfs*11)
65	357.8	138.6	5	BRCA2: c.3545_3546del p.(Phe1182ins*)
66	260.0	145.7	5	BRCA2: c.5946del p.(Ser1982Argfs*22)
67	534.9	244.0	5	BRCA2: c.8278_8279ins71 p.(Gly2760delinsVal*)
68	495.2	138.3	5	BRCA2: c.6486_6489del p.(Lys2162Asnfs*5)
69	411.6	167.7	5	BRCA2: c.5609_5610delinsAG p.(Phe1870*)
70	307.7	134.6	5	BRCA2: c.8755-2A>G
71	340.0	185.3	5	BRCA2: c.1813dup p.(Ile605Asnfs*11)
73	312.7	84.5	5	BRCA2: c.5682C>G p.(Tyr1894*)
74	216.0	90.6	5	BRCA2: c.7758G>A p.(Trp2586*)
75	316.2	89.7	5	BRCA2: c.7758G>A p.(Trp2586*)
76	344.1	137.7	5	BRCA2: c.658_659del p.(Val220Ilefs*4)
77	396.0	121.3	5	BRCA2: c.8323dup p.(Met2775Asnfs*7)
78	461.1	128.9	5	BRCA2: c.2808_2811del p.(Ala938Profs*21)
79	455.2	127.2	5	BRCA2: c.506del p.(Lys169Serfs*3)
80	366.2	141.3	5	BRCA2: c.7913_7917del p.(Phe2638ins*)
81	311.1	109.4	5	BRCA2: c.755_758del p.(Asp252Valfs*24)
82	254.6	122.1	5	BRCA2: c.3847_3848del p.(Val1283Lysfs*2)
84	208.7	153.3	5	BRCA2: c.5645C>A p.(Ser1882*)
85	510.4	133.1	5	BRCA2: c.5682C>G p.(Tyr1894*)
86	388.4	151.3	5	BRCA2: c.6275_6276del p.(Leu2092Profs*7)
87	502.5	118.5	5	BRCA2: c.2808_2811del p.(Ala938Profs*21)
255	339.2	192.8	5	BRCA2: c.5238dup p.(Asn1747*)
258	271.2	121.5	5	BRCA2: c.9118-2A>G
259	324.4	107.7	5	BRCA2: c.7069_7070del p.(Leu2357Valfs*2)
261	376.3	120.9	5	BRCA2: c.9371A>T p.(Asn3124Ile)
262	421.8	119.1	5	BRCA2: c.6331_6332del p.(Lys2111Glufs*17)
263	400.4	155.5	5	BRCA2: c.2489dup p.(Asn830Lysfs*3)
264	556.3	112.9	5	BRCA2: c.5213_5216del p.(Thr1738Ilefs*2)
265	500.1	176.9	5	BRCA2: c.9371A>T p.(Asn3124Ile)
266	487.2	139.1	5	BRCA2: c.1813dup p.(Ile605Asnfs*11)
267	435.5	145.6	5	BRCA2: c.3847_3848del p.(Val1283Lysfs*2)

269	410.2	185.4	5	BRCA2: c.1813dup p.(Ile605Asnfs*11)
270	374.5	203.6	5	BRCA2: c.1813dup p.(Ile605Asnfs*11)
271	439.8	249.5	5	BRCA2: c.4593dup p.(Val1532Serfs*2)
272	453.8	151.4	5	BRCA2: c.8486A>G p.(Gln2829Arg)
273	222.6	118.8	5	BRCA2: c.5645C>A p.(Ser1882*)
274	353.1	106.1	5	BRCA2: c.1925del p.(Ser642Leufs*2)
275	366.1	129.1	5	BRCA2: c.658_659del p.(Val220Ilefs*4)
276	350.7	100.1	5	BRCA2: c.9474del p.(Phe3159Serfs*4)
277	241.2	138.2	5	BRCA2: c.4284dup p.(Gln1429Serfs*9)
279	576.0	93.6	5	BRCA2: c.5800C>T p.(Gln1934*)
280	370.2	159.1	5	BRCA2: c.7878G>C p.(Trp2626Cys)
281	400.1	97.9	5	BRCA2: c.8394_8396delinsAA p.(Arg2799Asnfs*22)
282	380.2	153.0	5	BRCA2: c.6546del p.(Glu2184Asnfs*8)
283	376.9	138.0	5	BRCA2: c.9382C>T p.(Arg3128*)
344	463.0	145.1	5	BRCA2: c.8243G>A p.(Gly2748Asp)
349	588.5	114.3	5	BRCA2: c.3500_3501del p.(Ile1167Asnfs*16)
350	382.5	88.0	5	BRCA2: c.67+1G>A
351	434.5	168.6	5	BRCA2: c.5645C>A p.(Ser1882*)
354	404.8	183.4	5	BRCA2: c.8130del p.(Ser2710Argfs*23)
355	487.8	162.8	5	BRCA2: c.4588A>T p.(Lys1530*)
356	617.3	139.6	5	BRCA2: c.9672dup p.(Tyr3225Ilefs*30)
357	442.6	176.0	5	BRCA2: c.5645C>A p.(Ser1882*)
358	585.7	175.2	5	BRCA2: c.4478_4481del p.(Glu1493Valfs*10)
359	469.6	124.4	5	BRCA2: c.631+2T>G
360	607.5	108.0	5	BRCA2: c.3847_3848del p.(Val1283Lysfs*2)
361	375.2	214.1	5	BRCA2: c.7069_7070del p.(Leu2357Valfs*2)
362	502.4	147.8	5	BRCA2: c.1929del p.(Arg645Glufs*15)
363	516.7	126.4	5	BRCA2: c.658_659del p.(Val220Ilefs*4)

Supplementary Table 2

Primers for ddPCR of human 45S rDNA.

Assay	Primer	Sequence (5'-3')[a]
28S rDNA	Forward	5’-AACGTGAGCTGGGTTTAG-3’
	Reverse	5’-CTCGTACTGAGCAGGATTAC-3’
	Probe	5’-/5HEX/TGGCAACAA/ZEN/CACATCATCAGT/3IABkFQ/-3’
TBP	Forward	5’-GATATGAGACTGTGGGTAAGT-3’
	Reverse	5’-GATCCTTTGAACACCCTAATG-3’
	Probe	5’-/56-FAM/ACAGAGATC/ZEN/ACTGCAGTTGC/3IABkFQ/-3’

Supplementary Table 3

Primers for deep bisulfite sequencing of the human rDNA promoter (UCE/CP) region.

**Primer**	**Sequence (5'-3')[a]**	**Amplicon length**	**Variant**	**Annealing Temp. (^{o}C)**	**No. of CpGs**
Forward	TATTYGGAGGTTTAATTTTTTTAG	239 bp	A/G	56°C	25
Reverse	TATATCCTAAAATTAACCAAAAAACCCC				
